# Id4 and FABP7 are preferentially expressed in cells with astrocytic features in oligodendrogliomas and oligoastrocytomas

**DOI:** 10.1186/1472-6890-5-6

**Published:** 2005-07-15

**Authors:** Yu Liang, Andrew W Bollen, M Kelly Nicholas, Nalin Gupta

**Affiliations:** 1 Department of Neurological Surgery, Brain Tumor Research Center, University of California, San Francisco, CA 94143, USA; 2 Department of Pathology, University of California, San Francisco, CA 94143, USA; 3 Department of Neurology, University of Chicago, Chicago, IL 60637, USA

## Abstract

**Background:**

Oligodendroglioma (ODG) and oligoastrocytoma (OAC) are diffusely infiltrating primary brain tumors whose pathogenesis remains unclear. We previously identified a group of genes whose expression was inversely correlated with survival in a cohort of patients with glioblastoma (GBM), and some of these genes are also reportedly expressed in ODG and OAC. We examined the expression patterns and localization of these survival-associated genes in ODG and OAC in order to analyze their possible roles in the oncogenesis of these two tumor types.

**Methods:**

We used UniGene libraries derived from GBM and ODG specimens to examine the expression levels of the transcripts for each of the 50 GBM survival-associated genes. We used immunohistochemistry and cDNA microarrays to examine expression of selected survival-associated genes and Id4, a gene believed to control the timing of oligodendrocyte development. The expression of FABP7 and Id4 and the survival of patients with ODG and OAC were also analyzed.

**Results:**

Transcripts of most survival-associated genes as well as Id4 were present in both GBM and ODG tumors, whereas protein expression of Id4 and one of the survival-associated genes, brain-type fatty acid-binding protein (FABP7), was present in cells with astrocytic features, including reactive and neoplastic astrocytes, but not in neoplastic oligodendrocytes. Id4 was co-expressed with FABP7 in microgemistocytes in ODG and in neoplastic astrocytes in OAC. Id4 and FABP7 expression, however, did not correlate with the clinical outcome of patients with ODG or OAC tumors.

**Conclusion:**

Expression of Id4 and some of our previously identified GBM survival-associated genes is present in developing or mature oligodendrocytes. However, protein expression of Id4 and FABP7 in GBM, ODG, and OAC suggests that this group of functionally important genes might demonstrate two patterns of expression in these glioma subtypes: one group is universally expressed in glioma cells, and the other group of genes is expressed primarily in neoplastic astrocytes but not in neoplastic oligodendrocytes. Differential protein expression of these two groups of genes in ODG and OAC may be related to the cellular origins and the histological features of the neoplastic cells.

## Background

Gliomas are the most common subgroup of primary brain tumors. The majority of gliomas are classified on the basis of the histological appearance of the neoplastic cells: astrocytoma (WHO grades I to IV), oligodendroglioma (ODG; WHO grades II to III), and oligoastrocytoma (OAC; WHO grades II to III). ODG consist of cells morphologically resembling oligodendrocytes, and OAC consist of a spectrum of neoplastic cells, some with oligodendroglial features and others with astrocytic features. ODG and OAC often recur at a higher pathological grade, either as anaplastic ODG or OAC (grade III), or astrocytoma grade IV (glioblastoma, GBM) [1]. The exact genetic basis of the progression of lower-grade oligodendroglial tumors to higher-grade tumors is unclear. Both ODG and OAC have significantly better prognosis than GBM, with survival times ranging from 3 to 6 years, compared to 1 year for GBM [1-3]. Several genetic alterations in ODG and OAC, particularly the allelic loss of chromosome arms 1p and 19q, are used as prognostic indicators to predict longer recurrence-free survival after radiation therapy and/or chemotherapy [4-6].

In a previous study, we used gene expression profiling of a panel of 29 GBM specimens to identify a group of 50 named genes, designated as survival-associated genes, whose increased expression was inversely correlated with patient survival [7]. The tumor specimens were derived from 25 GBM tumors; in 4 of these specimens, 2 distinct regions from each tumor were analyzed. A two-step algorithm was used to identify the survival-associated genes [7]. First, by analyzing patient survival in relation to gene expression, a cluster of approximately 500 genes was identified whose increased expression in aggregate best inversely correlates with patient survival. This cluster of genes may represent a group of transcriptionally co-regulated genes, so it is plausible that they are involved in related biological processes. In the second step, we further selected 50 genes within this cluster that showed significantly greater variation in expression between different tumors than between the paired samples from the 4 specimens in which two distinct regions were analyzed. This subset of 50 genes is more likely to encode intrinsic properties related to the behavior and prognosis of individual GBM tumors. The prognostic value of one gene, brain-type fatty acid-binding protein (FABP7), was validated using two independent sets of GBM specimens [7].

The same microarray analyses also demonstrated that there was increased expression of the 50 survival-associated genes in ODG and OAC tumors compared to normal brain tissue [7]. This observation suggests that overexpression of the survival-associated genes may be common to different glioma subtypes. Expression of other genes in the group, such as *Ptprz1 *[8], *Bcan *[9], and *Crmp5 *[10], is also detected in oligodendrocytes in developing or injured brains, but their expression has not been reported in ODG and OAC. Two genes in the group, *Olig1 *and *Olig2*, were of particular interest since OLIG1 and OLIG2 are present in neural progenitor cells and oligodendrocytes but not in astrocytes and neurons. *Olig1 *and *Olig2 *encode basic helix-loop-helix (bHLH) transcription factors that play a central role in oligodendrocyte development in the central nervous system [11,12]. *Olig1/Olig2*-null mice show complete failure of oligodendrocyte development, and the precursor cells destined for an oligodendrocyte fate differentiate instead into astrocytes [12]. An initial report suggested that OLIG1 and OLIG2 might be markers for identification of oligodendroglial tumors [13]. More recent work, however, has demonstrated that OLIG1 and OLIG2 are expressed in all glioma subtypes including astrocytoma, ODG, and OAC [14,15].

In the group of 50 survival-associated genes, we did not identify any gene that has been previously reported to be a target of OLIG1 or OLIG2, or encode any protein known to interact with OLIG1 and OLIG2. However, in the larger cluster of 500 genes, *Id4*, a member of the helix-loop-helix (HLH) transcription factor family, was present. Id proteins dimerize with bHLH factors to prevent transcriptional induction of downstream target genes, and regulate a variety of functions in normal and neoplastic cells [16]. Several lines of evidence suggest the importance of Id4 in oligodendrocyte development. Id4 is expressed in oligodendrocyte precursor cells and is thought to control the timing of oligodendrocyte differentiation [17]. Enforced expression of Id4 stimulates proliferation and blocks differentiation of oligodendrocyte precursor cells [17]. Id4 was recently found to directly interact with OLIG1 and OLIG2 in neural progenitor cells and to mediate the inhibitory effects of bone morphogenetic protein-4 (BMP-4) on oligodendroglial differentiation [18]. OLIG1/2 and Id4 are co-expressed in neural progenitor cells, in which OLIG1 and OLIG2 are localized in the nucleus whereas Id4 is localized in the cytoplasm; when Id4 expression is induced after BMP-4 treatment, OLIG1/2 localized predominantly to the cytoplasm and co-localizes with Id4 [18]. Despite this evidence *in vitro*, the expression of Id4 as it relates to OLIG1 and OLIG2 expression in ODG and OAC has not been reported.

In this study, we analyzed *in silico *the levels of expression for each survival-associated gene in the UniGene libraries derived from GBM and ODG specimens. We then examined the expression of Id4 in tissue samples from ODG and OAC using immunohistochemistry and found that, in contrast to the patterns of expression for OLIG1 and OLIG2, Id4 is preferentially expressed in cells of astrocytic lineage in ODG and OAC tumors, which is similar to the expression patterns of FABP7 (unpublished data). The differential expression of Id4 and FABP7 in cells of astrocytic phenotype in GBM and oligodendroglial tumors suggests that specific transcription factors functioning in cell-fate determination during development might also determine the development of histopathologically distinct glioma subtypes.

## Methods

### *In silico *analysis

UniGene libraries for GBM and ODG were used to examine the normalized expression of the 50 survival-associated genes. Library derived from OAC was not available at the time of analysis. UniGene assigns all human Expressed Sequence Tag (EST) sequences that meet minimal standards of quality to distinct "clusters". Each cluster contains sequences that represent a unique human expressed gene, and related information such as the tissue types from which the libraries generating the sequences were prepared [19]. The normalized expression for each survival-associated genes as well as for Id4 in tissues was recorded from the SOURCE database . The normalized expression of a gene represents the relative expression level (in percentile) in different tissues and is normalized for the number of EST clones from all libraries reported by UniGene.

### Tissue specimens

Paraffin-embedded and frozen specimens were obtained from the Brain Tumor Research Center Tissue Bank at the University of California, San Francisco after approval from the Committee on Human Research.

### Microarray analysis

Sample preparation and microarray methods followed those of our previous study [7]. Briefly, total RNA was extracted from frozen tissue specimens using Trizol (Invitrogen; Carlsbad, CA) followed by mRNA purification using FastTrack (Invitrogen). Messenger RNA was reverse transcribed to cDNA and directly labeled with Cy dyes (Amersham Biosciences; Piscataway, NJ) before hybridization. Other detailed protocols can be found in web supplement .

### Antibodies

FABP7 antiserum was a gift from Dr. Nathaniel Heintz (Rockefeller University, New York, NY). A dilution of 1 to 400 was used for both immunostaining and migration assays, and a dilution of 1 to 500 was used for immunoblotting. Dilutions of antibodies against glial fibrillary acidic protein (GFAP) (ICN; Costa Mesa, CA) and Id4 (Santa Cruz Biotechnology, Santa Cruz, CA) for immunohistochemistry were 1:1000 and 1:100, respectively. A dilution of 1 to 100 of the Id4 antibody was used for immunoblotting. Peroxidase-conjugated and biotinylated secondary antibodies were obtained from Vector Laboratories (Burlingame, CA).

### Western blot analysis

The protein fraction of frozen tissue specimens was purified by isopropanol precipitation after removing total RNA and genomic DNA using Trizol, washed several times in 0.3 M guanidine hydrochloride in 95% ethanol, and resuspended in 1% SDS. The protein concentration of each sample was quantitated by using a D_c _Protein Assay Kit (Bio-Rad; Hercules, CA), and equal amounts of protein for each sample were separated by SDS-PAGE and transferred to nitrocellulose membranes (Bio-Rad), blocked with 10% skim milk, incubated with specific antibodies, and visualized using a Super Signal West Pico Chemiluminescent kit (Pierce; Rockford, IL).

### Immunohistochemistry

All frozen tissue sections used for immunohistochemistry were fixed in 4% formaldehyde, treated with H_2_O_2_, blocked with normal serum, incubated with primary antibodies at 4°C overnight or at room temperature (RT) for 2 hours, and incubated with biotinylated secondary antibody and peroxidase-labeled streptavidin at RT for 30 minutes. The DAB Reagent kit was used to visualize the immunoreactivity (KPL; Gaithersburg, MA). Staining of paraffin-embedded sections followed the same protocol, except for prior de-waxing and antigen retrieval using microwave heating.

### Statistical analyses

All statistical analyses used SPSS for Windows (Release 11.5.0). Comparison of Id4 mRNA levels in gliomas was analyzed using Student's t test. Correlation of Id4 and FABP7 expression with patient survival was analyzed using the Cox proportional hazards regression. A p value ≤0.05 was considered statistically significant for all tests.

## Results

### Transcripts of most survival-associated genes identified in glioblastomas are also detected in oligodendrogliomas

In our previous microarray analyses, we found elevated expression of the GBM survival-associated genes in ODG and OAC as compared to normal brain specimens [7]. However, expression of these 50 survival-associated genes in ODG and OAC tumors has not been previously studied except *Olig1 *and *Olig2 *[14]. To corroborate our previous findings, we examined *in silico *the normalized expression for each survival-associated gene as well as for *Id4 *in UniGene libraries derived from GBM and ODG tumors. We found that, among the 50 genes, EST clones from 36 of the genes (72%) were present in GBM libraries, and clones from 30 of them (83%) were detected in libraries derived from ODG specimens (Table 1). As expected, significant numbers of EST clones representing *Olig1*, *Olig2*, and *Id4 *appeared in libraries from ODG. This result indicates that, at least at the mRNA level, most of the survival-associated genes identified in GBM can also be detected in ODG, which correlates with the results from our previous microarray analyses.

**Table 1:** Normalized expression of the survival-associated genes in GBM and oligodendrogliomas

Clone ID	Gene Name	GBM (%)^1^	Oligodendroglioma (%)
IMAGE:222457	ARC	56.16	3.53
IMAGE:383898	ARHN	5.1	28.36
IMAGE:1416420	ASCL1	5.79	7.39
IMAGE:32687	BCAN	12.5	38.22
IMAGE:290213	CACNG4	7.3	24.99
IMAGE:249688	CCND2	1.85	0.8
IMAGE:295116	CHRFAM7A	ND^2^	ND
IMAGE:220096	CNGB1	ND	ND
IMAGE:26295	CNR1	16.02	ND
IMAGE:248485	CRB1	ND	9.04
IMAGE:26505	CRMP5	14.59	5.42
IMAGE:41720	CRMP5	14.59	5.42
IMAGE:43771	CROC4	29.82	14.65
IMAGE:301104	CSPG5	8.02	16.41
IMAGE:33854	DBCCR1L	ND	0.01
IMAGE:951022	DCX	19.01	3.78
IMAGE:361688	DPP6	2.18	14.1
IMAGE:81320	ETV1	1.76	4.17
IMAGE:279195	FABP7	ND	1.44
IMAGE:345626	FABP7	ND	1.44
IMAGE:773976	FABP7	ND	1.44
IMAGE:75759	FLJ10748	18.43	1.17
IMAGE:669379	GLCCI1	ND	0.44
IMAGE:49987	GRIA2	1.4	3.13
IMAGE:730016	KCNJ10	23.1	2.88
IMAGE:50939	KIAA0523	2.05	4.62
IMAGE:192543	KIAA0773	2.48	15.04
IMAGE:927112	KIAA0773	2.48	15.04
IMAGE:397495	LRRTM2	ND	ND
IMAGE:787860	MGC45428	0.34	0.52
IMAGE:838478	NCALD	1.22	1.92
IMAGE:838668	NSE1	0.46	0.3
IMAGE:743187	NUDT10	ND	ND
IMAGE:41214	OLIG1	2.92	33.23
IMAGE:26884	OLIG2	13.58	34.21
IMAGE:287468	PCDH17	ND	0.95
IMAGE:166934	PCDHGC3	9.46	8.46
IMAGE:753071	PHLDA1	4.27	3.71
IMAGE:866702	PTPN13	ND	ND
IMAGE:30175	PTPRO	ND	1.12
IMAGE:785148	PTPRZ1	5.34	8.2
IMAGE:161436	PTPRZ1	5.34	8.2
IMAGE:283976	PURG	13.16	ND
INAGE:1636226	SALL1	ND	20.31
IMAGE:207087	SCN	5.43	1.96
IMAGE:179534	SCNQ2	3.55	20.51
IMAGE:361659	SGEF	2.27	ND
IMAGE:357220	SHC3	ND	ND
IMAGE:45877	SLC29A4	ND	1.44
IMAGE:38015	SLITRK2	ND	ND
IMAGE:758266	THBS4	ND	1.19
IMAGE:34204	TRIM9	6.54	4.31
IMAGE:951705	ZCCHC6	0.76	ND
IMAGE:359684	ZDHHC22	32.39	17.7

	Id4	2.1	8.29

^1 ^Normalized expression level of each gene is defined as the number of EST clones detected in tumor libraries (GBM or oligodendrogliomas) divided by the number of clones detected in all libraries reported by UniGene.

^2 ^ND, not detected.

### Id4 protein is readily detected in glioblastomas and oligoastrocytomas but not in oligodendrogliomas

**Figure 1:**
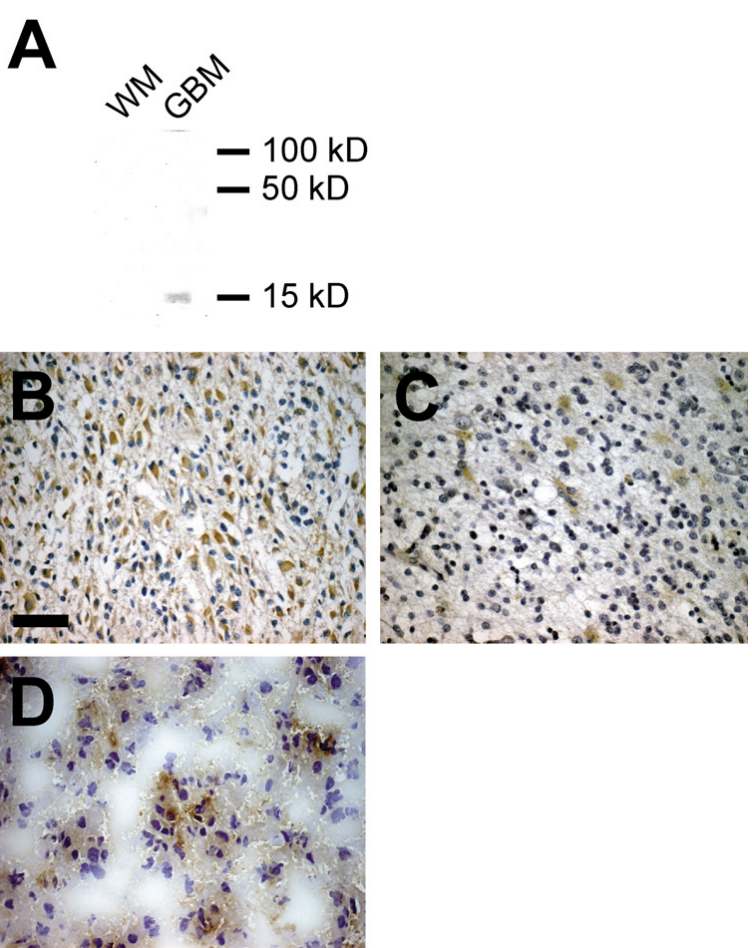
Id4 is preferentially expressed in cells of astrocytic lineage. The specificity of anti-Id4 antibodies was confirmed with immunoblotting by using Trizol extracts of a GBM specimen and white matter (WM) from a normal subject (A). Cytoplasmic Id4 immunoreactivity was prominent in most GBM examined (B). In oligodendrogliomas, neoplastic oligodendrocytes were essentially negative for Id4, whereas some reactive astrocytes could be found Id4-positive (C). Id4 immunoreactivity could also be seen in the astrocytic component of some oligoastrocytomas (D). Bar in B, 50 μm.

Because the *in silico *analysis of tumor specimens cannot distinguish the cellular source of expression, we used immunohistochemistry to localize the expression of Id4 in GBM and oligodendroglial tumors. The specificity of Id4 antibodies was demonstrated by detection of a 15 kD band on immunoblot using Trizol extracts of GBM and normal brain specimens (Figure 1A). Id4 immunoreactivity was found in 10% to 100% of tumor cells in 12 of the 13 GBM specimens we examined (92%), and Id4 in both nucleus and cytoplasm could be detected in immunoreactive cells (Figure 1B). Among the 21 ODG tumors examined, expression of Id4 was seen in some reactive astrocytes but was essentially negative in neoplastic oligodendrocytes (Figure 1C), while unequivocal Id4 staining was observed in some neurons associated with tumor cells (data not shown). We also examined 10 OAC specimens, all of which had GFAP-positive neoplastic cells and 5 of them (50%) had detectable Id4 immunoreactivity in GFAP-positive areas (Figure 1D).

**Figure 2:**
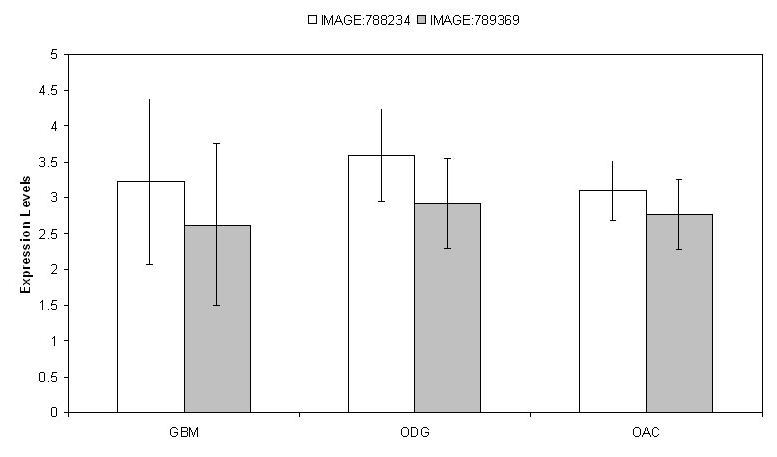
Microarray analysis of Id4 mRNA in glioma tissues that have been examined with immunohistochemistry. The expression levels of Id4 were represented by the log 2-transformed ratio of the fluorescence signal in tumor to the signal in hybridization reference. Two IMAGE clones (788234 and 789369) corresponding to different regions of the *Id4 *gene with interpretable data were selected for analysis. All three tumor types examined in this analysis demonstrated similar levels of Id4 mRNA (p values between 0.33 and 0.75).

Our *in silico *and immunohistochemistry data showed that Id4 mRNA had comparable levels in UniGene libraries derived from GBM and ODG specimens (Table 1), while Id4 protein was detected only in neoplastic astrocytes but not in neoplastic oligodendrocytes (Figure 1). To confirm the discrepancy of the Id4 expression between mRNA and protein levels in ODG tumors, we examined expression of Id4 mRNA in selected GBM (N = 7), ODG (N = 3), and OAC specimens (N = 3) from a previous microarray study [7]; all these tumor specimens had been examined for Id4 expression with immunohistochemistry and showed Id4 immunoreactivity in neoplastic astrocytes but not in neoplastic oligodendrocytes. Unlike protein expression, we found that Id4 mRNA had similar levels in specimens derived from all three tumor types examined in this analysis (p = 0.6, Figure 2).

### Id4 and FABP7 are co-expressed in microgemistocytes of oligodendrogliomas and in neoplastic astrocytes of oligoastrocytomas

Although Id4 has an expression pattern similar to OLIG1 and OLIG2 in GBM, Id4 protein was preferentially detected in reactive and neoplastic astrocytes but not in neoplastic oligodendrocytes. We then examined whether this difference occurred with other genes in the survival-associated group. We chose to study expression of FABP7 in ODG and OAC and found that, similar to Id4, FABP7 was expressed in OAC but not in the neoplastic oligodendrocytes (unpublished data). We therefore examined whether both genes are co-expressed in ODG and OAC tumors.

**Figure 3:**
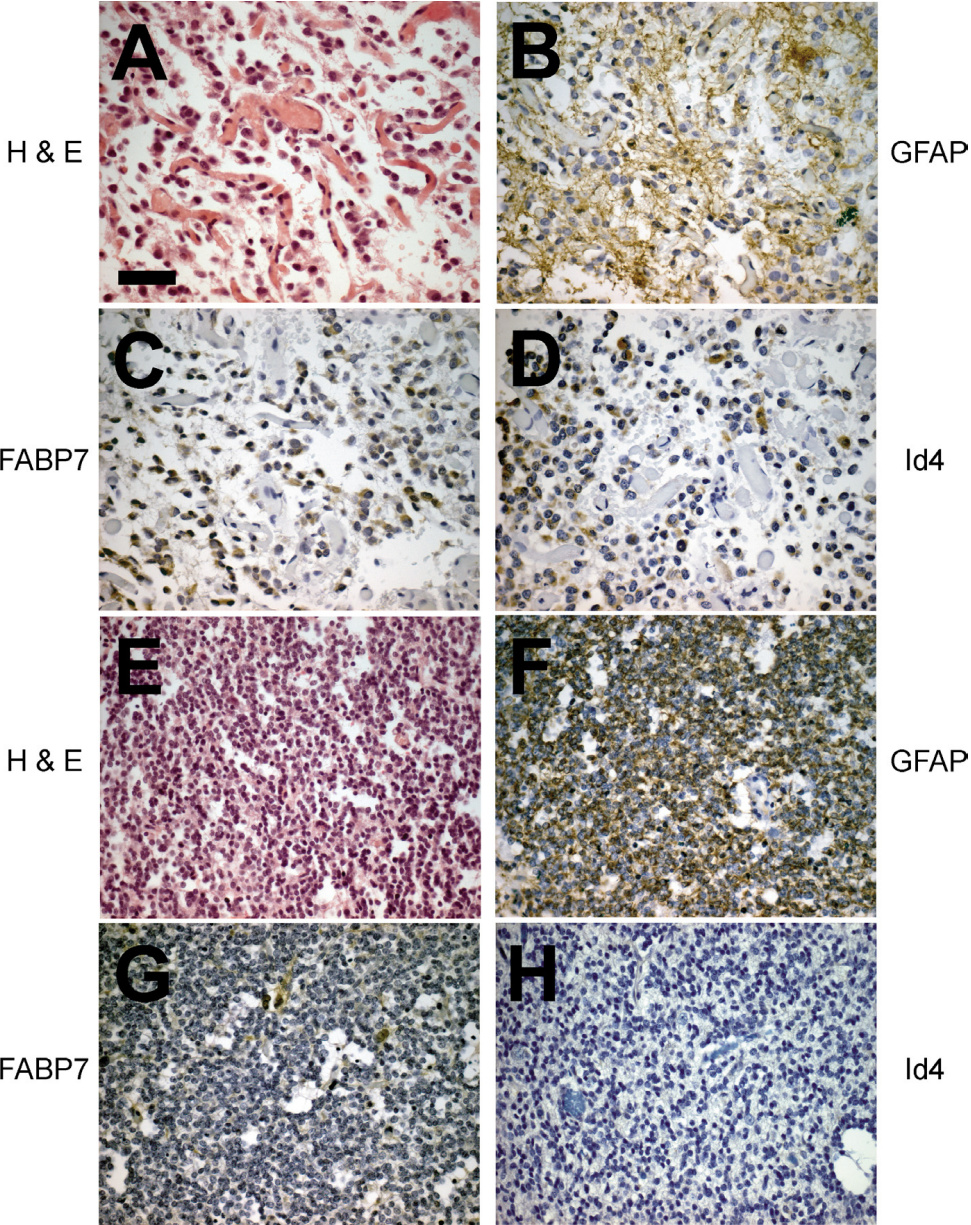
FABP7 and Id4 are co-expressed in microgemistocytes in oligodendrogliomas. H & E staining showed typical morphology of microgemistocytes with eosinophilic cytoplasm (A) with strong GFAP immunoreactivity (B). In consecutive sections, cytoplasmic FABP7 (C) and Id4 (D) staining was detected in the same group of cells. However, microgemistocytes are negative for both FABP7 and Id4 in some oligodendrogliomas. H & E (E) and GFAP (F) staining showed the presence of microgemistocytes that were essentially negative for FABP7 (G) and Id4 (H). Some FABP7-positive cells were reactive astrocytes (G). Bar in A, 50 μm.

**Figure 4:**
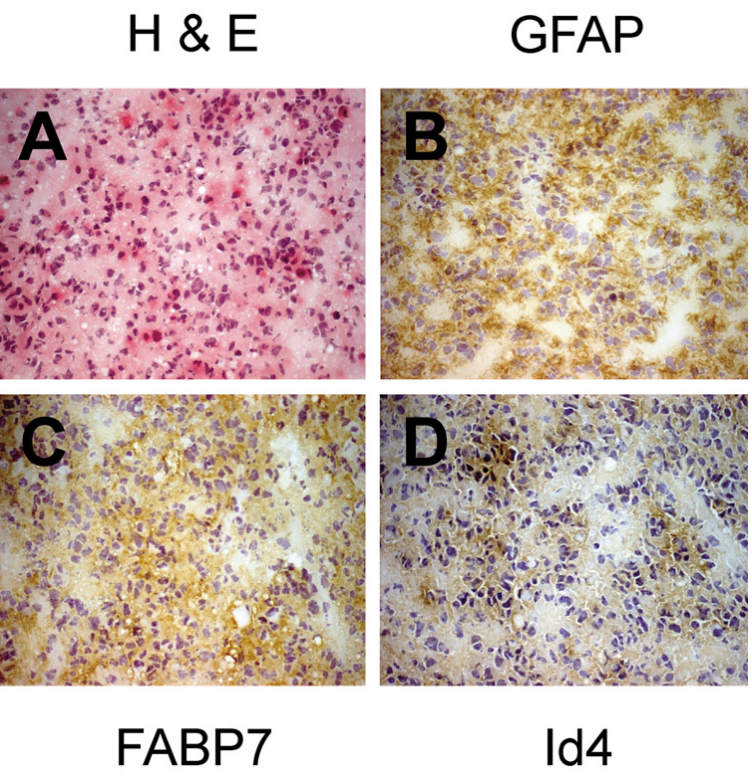
FABP7 and Id4 are co-expressed in astrocytic component of oligoastrocytomas. H & E staining demonstrated the presence of neoplastic astrocytes with eosinophilic cytoplasm and elongated nuclei (A) that were also GFAP-positive (B). The same population of cells was positive for both FABP7 (C) and Id4 (D) in consecutive sections of the frozen specimen. The scale of the photomicrographs is the same as in Figure 1B.

Microgemistocytes are a small subset of neoplastic cells that may be seen in ODG tumors, but the exact cellular origin of this type of cells is still controversial. Six ODG specimens had an easily discernible group of GFAP-positive microgemistocytes (Figure 3A and 3B), and in 4 of these specimens both FABP7 and Id4 immunoreactivity was present in the cytoplasm (Figure 3C and 3D). No Id4 immunoreactivity was detected in FABP7-negative microgemistocytes (Figure 3E to 3H). Among the 10 OAC specimens examined, 8 were positive for FABP7, and 5 of these 8 specimens had detectable Id4 immunoreactivity. In this subgroup of 5 OAC specimens (Figure 4A), GFAP (Figure 4B), FABP7 (Figure 4C), and Id4 (Figure 4D) were observed in the same population of neoplastic astrocytes.

### Expression of Id4 and FABP7 in relation to the clinical outcome of patients with oligodendrogliomas and oligoastrocytomas

Given the findings that nuclear FABP7 immunoreactivity predicts the outcome of patients with GBM, and that Id4 and FABP7 are co-expressed in OAC and in microgemistocytes of ODG, we tested whether expression of Id4 and FABP7 might have prognostic value for patients with ODG or OAC. Among the tumor specimens on which we performed immunohistochemistry, we analyzed 15 ODG (grade III) and 10 OAC (1 grade II and 9 grade III) for which we could obtain clinical information about the patients (Table 2). The median age for this group of patients with ODG and OAC was 51 and 45 years old, respectively. The follow-up period from the time of initial diagnosis ranged from 3 to 9 years. Although they were not treated under the same protocol, all had surgical resections and received both radiotherapy and alkylating chemotherapy.

**Table 2:** Summary of clinical data of patients with ODG and OAC, and immunohistochemistry of their tumors.

Tumor ID	Diagnosis	Alive/Dead^4^	Survival Time (year)^5^	GFAP	FABP7	Id4
2650	ODG III^1^	A	9.12	- (MG+)^6^	- (MG+)	- (MG+)
2963	ODG III	A	7.28	- (MG+)	- (MG+)	- (MG+)
3081	ODG III	A	6.97	- (MG+)	- (MG+)	- (MG+)
3474*	ODG III	A	5.67	+ (?)^7^	-	-
3492	ODG III	D	5.29	-	-	-
3551*	ODG III	A	5.42	NI^8^	-	-
3607	ODG III	D	3.91	-	-	-
3651*	ODG III	A	5.09	-	-	-
3767	ODG III	A	4.73	-	-	-
3768	ODG III	A	4.73	-	-	-
3815	ODG III	A	4.59	- (MG+)	-/+^9 ^(MG-)	- (MG-)
4115	ODG III	A	3.64	- (MG+)	- (MG+)	- (MG+)
4142	ODG III	A	3.55	- (MG+)	- (MG-)	- (MG-)
4167	ODG III	A	3.48	-/+	-/+	-
4253	ODG III	A	3.26	-	-	-

2450	OAC III^2^	A	9.9	+	+ (cyto/nuc)^10^	+ (cyto)
2459	OAC III	A	9.9	+	+ (cyto/nuc)	+ (cyto)
2533*	OAC III	A	6.55	+	+ (cyto)	-
2609*	OAC III	A	9.27	+	+ (cyto/nuc)	-
2622*	OAC III	D	5.75	+	-	-
2668	OAC III	D	8.97	+	+ (cyto/nuc)	+ (cyto)
3304	OAC III	A	6.27	+	+ (cyto)	+ (cyto)
3378	OAC III	A	5.97	+	+ (cyto/nuc)	+ (cyto/nuc)
3729	OAC II^3^	A	4.85	+	+ (cyto)	-
4141	OAC III	A	3.55	+	-	-

^1 ^ODG III, oligodendroglioma, grade III.

^2 ^OAC III, oligoastrocytoma, grade III.

^3 ^OAC II, oligoastrocytoma, grade II.

^4 ^Alive or dead of the patients was determined by the date of the most recent follow-up (9/30/04).

^5 ^The date of diagnosis to 9/30/04 was defined as the survival time for patients who were alive.

^6 ^Positive in microgemistocytes (MG).

^7 ^This was a frozen section with 50% of tumor cells positive for GFAP, but H & E did not show microgemistocytes; the result was therefore questionable.

^8 ^This was a frozen section with lots of freezing artifect; the result was not interpretable.

^9 ^A small group (<5%) of tumor nuclei were positive.

^10 ^Both cytoplasm and nuclei were positive.

* Specimens that have been analyzed by cDNA microarrays.

Two of the 15 patients with ODG have died, and their tumors were negative for Id4/FABP7 immunoreactivity (Table 2). Two of the 10 patients with OAC have died; one tumor had no detectable Id4 and FABP7 immunoreactivity, while the other one showed a similar degree of cytoplasmic/nuclear FABP7 and cytoplasmic Id4 staining to the other 4 specimens derived from patients who were still alive (Table 2). There were no characteristics that distinguished the living subjects from those diseased. We used Cox regression to analyze the correlation of Id4 and FABP7 immunoreactivity with patient survival by the presence of immunoreactive microgemistocytes (for stratifying ODG patients), or by the immunoreactivity in nucleus alone or in both nucleus and cytoplasm (for stratifying OAC patients). Our limited data showed that expression of Id4 and FABP7 did not correlate with the survival of patients with ODG or OAC tumors (Table 3).

**Table 3:** Cox regression analyses of FABP7 and Id4 immunoreactivity in relation to the survival of ODG and OAC patients

Tumor	Markers	Immunoreactivity Status	no. of cases	*p*
ODG	FABP7 & Id4	MG+ vs. MG-	4 vs. 11	0.524
	FABP7	cyto/nuc+ or cyt+ vs. -	8 vs. 2	0.859
OAC		nuc+ vs. nuc-	5 vs. 5	0.581
	Id4	cyto/nuc+ or cyt+ vs. -	5 vs. 5	0.573

## Discussion

Oligodendroglial tumors are the third most common type of primary brain tumor. They have a diffusely infiltrative growth pattern similar to astrocytic tumors but tend to have a better prognosis. The exact genetic basis for the development of gliomas with astrocytic, oligodendroglial, or mixed (oligoastrocytic) phenotypes is unknown. However, it has been recognized that specific genetic backgrounds do affect the clinical outcome of patients with oligodendroglial tumors. A subgroup of oligodendroglial tumors with allelic loss of chromosome arms 1p and 19q respond better to treatment [20]. Several genetic aberrations frequently detected in GBM, such as mutations in the *p53 *gene, *Egfr *overexpression or amplification, and deletion of the *p16/Cdkn2A *gene, are also associated with poor prognosis in both ODG and OAC. Defining the genetic features of these glial neoplasms has both diagnostic and potentially therapeutic benefits. Previously, we identified a group of genes that stratifies patients with histologically identical GBM into two groups with markedly different survival [7]. The expression of FABP7, in particular, demonstrated a strong inverse correlation with patient survival, and may be involved in the pathogenesis of GBM (unpublished data). Our data suggested that this group of genes that includes *Olig1 *and *Olig2 *might be also expressed in ODG and OAC [7]. Therefore, we hypothesized that our group of survival-associated genes originally identified in GBM might also be associated with the pathobiology of oligodendroglial neoplasms.

We examined *in silico *the expression of the transcripts for each survival-associated gene in UniGene libraries derived from GBM and ODG tumors, and most could be identified in both tumor types. The transcripts of the *Fabp7 *gene were not detected in the GBM libraries from UniGene (Table 1). This might be because FABP7 immunoreactivity was observed in only half to two-thirds of the GBM specimens as shown in our previous studies [7], and the number of libraries reported by UniGene may not be sufficient to "capture" all potentially positive tumors. Another limitation of this type of analysis is that the cellular source for positive gene expression cannot be determined. For this reason, we validated our analysis by examining specific genes at the tissue level using immunohistochemistry.

Our data demonstrate that Id4 and FABP7 proteins are primarily localized in cells with astrocytic features but not in neoplastic oligodendrocytes. Therefore, the expression of survival-associated genes in ODG and OAC can be divided into at least two subgroups; genes such as *Olig1 *and *Olig2 *that are expressed in all glial tumors, and genes such as *Fabp7 *that are restricted to astrocytes. This division is supported by our immunohistochemical analysis of another survival-associated gene, *Ptprz1*, which has an expression pattern in ODG and OAC similar to Id4 and FABP7 (unpublished data).

Examining the biological roles of OLIG1, OLIG2, and Id4 in developing central nervous system provides a plausible explanation for these varying patterns of gene expression. OLIG1 and OLIG2 play a central role in oligodendrocyte development, as illustrated by their expression in fully differentiated oligodendrocytes [21], the failure of oligodendrocyte differentiation in *Olig1*/*Olig2*-knockout mice [12], and the expansion of oligodendroglial cells *in vivo *following overexpression of OLIG1 and OLIG2 [22,23]. Oligodendrocyte development exemplifies the principle of 'switching' of cell fate by alteration of transcription-factor expression levels. For example, overexpression of BMP-4 in mice results in a significant decrease in the number of oligodendrocytes, accompanied by an increase in astrocytes [24], coincident with the gradual decline of OLIG1 and OLIG2 [18]. Ectopic expression of Id4 in neural progenitor cells recapitulates the effect of BMP-4 by blocking oligodendrocyte maturation, stimulating cell-cycle progression of oligodendrocyte precursor cells, and promoting astrocyte formation [17,18]. Id4 expression decreases when the precursor cells are induced to differentiate into oligodendrocytes [17]. OLIG1, OLIG2, and Id4 are co-expressed in neural progenitor cells but in different subcellular compartments (OLIG1 and OLIG2 are localized in the nucleus and Id4 is localized in the cytoplasm). BMP-4 treatment induces Id4 expression along with translocation of OLIG1 and OLIG2 to the cytoplasm; therefore, it was proposed that cytoplasmic Id4 must reach a critical level to effectively sequester OLIG1 and OLIG2 to prevent their nuclear translocation, and subsequent specification of the cell fate [18].

Both astrocytomas and oligodendroglial tumors in humans demonstrate diffuse expression of OLIG1 and OLIG2, although expression of OLIG2 is lower and increasingly variable and heterogeneous in GBM as compared to ODG tumors [14]. Conversely, Id4 is negative in neoplastic oligodendrocytes, positive in neoplastic astrocytes in half of the OAC specimens examined, and positive in almost all GBM specimens. This pattern of Id4 expression parallels its expression in neural progenitor cells during their commitment to either an oligodendrocytic or astrocytic lineage. Based on these observations, one possibility is that astrocytomas and oligodendroglial tumors may arise from a common cell type with features similar to the OLIG1/2-positive neural progenitor cells. Increased expression of Id4 by growth-factor stimulation or genetic mutation may switch transformed cells towards an astrocytic phenotype, whereas failure of Id4 expression would give rise to pure ODG.

The astrocytic component of OAC may represent a focal area of Id4 expression in tumor subpopulations occurring at later stages after tumor initiation, leading to the co-expression of Id4 with OLIG1 and OLIG2 in neoplastic astrocytes and lack of Id4 expression in neoplastic oligodendrocytes. This potential mechanism for the presence of mixed cell populations in OAC is consistent with genetic analyses demonstrating that astrocytic and oligodendroglial components within an oligoastrocytoma might be of monoclonal origin [25]. Furthermore, the magnitude and the timing of Id4 induction could explain the heterogeneity of OAC, in that one group is genetically related to ODG, and the other one related to astrocytomas [26,27]. However, the astrocytic component in OAC may have a different transitional nature from the GFAP/Id4/FABP7-positive cell types in ODG, since a recent study found that no OLIG1 expression is seen in GFAP-positive microgemistocytes in ODG tumors [28]. In this study, we did not find a correlation between Id4 and FABP7 expression and the clinical outcome of patients with OAC, but it may be of interest to repeat the analyses in a larger cohort of astrocytoma-like OAC as defined in the study by Maintz and others [26,27].

In conclusion, we show that Id4 expression is present primarily in neoplastic astrocytes in both oligodendroglial tumors and GBM, which is in contrast to the expression patterns of *Olig1 *and *Olig2*, two survival-associated genes originally identified in GBM tumors. Two other survival-associated genes, *Fabp7 *and *Ptprz1*, show expression patterns similar to Id4. Based on the function of Id4, OLIG1, and OLIG2 during normal development, our data suggest that Id4 and potentially other genes such as *Fabp7 *and *Ptprz1 *might account for the formation of distinct glioma subtypes during oncogenic progression.

## Conclusion

Id4 expression is present primarily in neoplastic astrocytes in both oligodendroglial tumors and GBM, which is in contrast to the expression patterns of OLIG1 and OLIG2, two survival-associated genes originally identified in GBM tumors. FABP7 show expression patterns similar to Id4, indicating that the survival-associated genes can be divided into at least two groups in ODG and OAC. The functions of Id4, OLIG1, and OLIG2 during normal development, and our data suggest that Id4 and potentially other proteins such as FABP7 and PTPRZ1 might account for the formation of distinct glioma subtypes during oncogenic progression.

## List of abbreviations

FABP7, brain-type fatty acid-binding protein; HLH, helix-loop-helix; GBM, glioblastoma; ODG, oligodendroglioma; OAC, oligoastrocytoma; WHO, World Health Organization.

## Competing interests

The author(s) declare that they have no competing interests.

## Authors' contributions

YL designed the study, performed the experiments, and wrote the manuscript. AWB participated in evaluating the immunohistochemistry. MKN participated in collecting clinical data of patients. NG participated in editing the manuscript and provided laboratory resources for experiments.

## Pre-publication history

The pre-publication history for this paper can be accessed here:


